# The Use of a Shelter Software ^a^ to Track Frequency and Selected Risk Factors for Feline Upper Respiratory Infection

**DOI:** 10.3390/ani5020161

**Published:** 2015-03-25

**Authors:** Ann Therese Kommedal, Denae Wagner, Kate Hurley

**Affiliations:** Koret Shelter Medicine Program, Center of Companion Animal Health, University of California, Davis, One Shield Avenue, Davis, CA 95616, USA; E-Mails: dcwagner@ad3.ucdavis.edu (D.W.); kfhurley@ucdavis.edu (K.H.)

**Keywords:** shelter medicine, cats, population management, herd health, feline upper respiratory infection, infectious disease, data quality

## Abstract

**Simple Summary:**

Feline upper respiratory infection is a common disease in animal shelters. Without monitoring, effective control and prevention is difficult. We looked at a software system ^a^ used in shelters across the United States to determine if it can be used to track URI frequency and risk factors in a population. Reports from the software system ^a^ were compared to data collected manually. This showed that data currently collected were not useful for tracking URI frequency and risk factors. However, potential exists to increase the practicality and usefulness of this shelter software system to monitor URI and other diseases.

**Abstract:**

**Objective**—Feline upper respiratory infection (URI) is a common, multi-factorial infectious disease syndrome endemic to many animal shelters. Although a significant cause of morbidity and mortality in shelter cats, URI is seldom formally monitored in shelter cat populations. Without monitoring, effective control and prevention of this often endemic disease is difficult. We looked at an integrated case management software system ^a^ for animal care organizations, widely used in shelters across the United States. Shelter staff routinely enter information regarding individual animals and disease status, but do not commonly use the software system to track frequency of disease. The purpose of this study was to determine if the software system ^a^ can be used to track URI frequency and selected risk factors in a population, and to evaluate the quality and completeness of the data as currently collected in a shelter. **Design (type of study)**—Descriptive Survey. **Animals (or Sample)**—317 cats in an animal shelter. **Procedures**—Reports from the software system ^a^ containing data regarding daily inventory, daily intake, animal identification, location, age, vaccination status, URI diagnosis and URI duration were evaluated. The reports were compared to data collected manually by an observer (Ann Therese Kommedal) to assess discrepancies, completeness, timeliness, availability and accuracy. Data were collected 6 days a week over a 4 week period. **Results**—Comparisons between the software system ^a^ reports and manually collected reports showed that 93% of inventory reports were complete and of these 99% were accurate. Fifty-two percent of the vaccination reports were complete, of which 97% were accurate. The accuracy of the software system’s age reports was 76%. Two-hundred and twenty-three cats were assigned a positive or negative URI diagnosis by the observer. The predictive value of the URI status in the software system ^a^ was below 60% both for positive and negative URI diagnosis. **Conclusions and Clinical Relevance**—data currently collected and entered into the software systems in the study shelter, was not useful for tracking URI frequency and risk factors, due to issues with both data quality and capture. However, the potential exists to increase the practicality and usefulness of this shelter software system to monitor URI and other diseases. Relevant data points, *i.e.*, health status at intake and outcome, vaccination date and status, as well as age, should be made mandatory to facilitate more useful data collection and reporting.

## 1. Introduction

Feline upper respiratory infection (URI) is a common, multi-factorial disease syndrome endemic in many animal shelters. Upper respiratory infections disseminate readily in the shelter environment. Acute URI often develops shortly after arrival to a shelter and is responsible for a significant loss of well-being for shelter cats. Chronic URI disease can develop in cats secondary to acute disease and may cause long-term health and welfare problems for the adopted cat [1]. Caring for cats with URI can create a significant financial drain on shelter resources and in some circumstances URI can even be considered a potentially “fatal disease”: many cats suffering from the condition are euthanized due to the risk of disease transmission to other shelter cats, the resources required to care for affected cats, and the reduced potential for affected cats to be adopted [2]. Developing effective management strategies to control feline URI is largely dependent on the availability and quality of data collection and must take into consideration both environmental and host factors. Several studies in animal shelters and catteries have shown that the risk factors for developing URI include age, number of days in the shelter, level of hygiene and larger number of cats [2,3,4,5].

Risk factors for URI and the efficacy of various strategies for control likely vary between shelters and over time. In order to permit recommendations tailored to an individual shelter population, data collection must be consistent and ongoing. Research methods employed to date for tracking URI in shelter cats have been prohibitively time intensive to permit routine use on an ongoing basis [4,5]. Resources for record-keeping in animal shelters are often limited and shelter managers may be uncertain as to what data are most pertinent. Shelter data collection systems have historically focused on intake and outcome information or individual animal tracking, resulting in a lack of useful data to tailor disease control programs, measure impacts of protocols or prevent misallocation of resources [6]. However, if data currently entered into existing software systems could be used to track risk factors and disease outcomes on a consistent basis, appropriate herd data collection and analysis in animal shelters should be possible without imposing a significant extra time burden on shelter staff.

We looked at an integrated shelter software case management system ^a^ for animal care organizations which is widely used in shelters across the United States. The requirements for data entry into the software system include both “mandatory” and “optional” components. Shelter staff members routinely assess and enter individual animal information such as date of entry, age, vaccination and health status.

The quality of these data can be assessed by their completeness, timeliness, availability and accuracy. Accuracy of data refers to the degree to which data correctly reflect the real world event being described. Systems for classification, for instance of health status and age, must be clearly understood by staff performing these assessment and used consistently in order to allow for accurate tracking and comparison over time. These data points must then be entered correctly and consistently into the data tracking system to allow for assessment.

The purpose of our study was to determine if the data routinely assessed and entered in the software system ^a^ were of sufficient quality to accurately track URI frequency and selected risk factors in one shelter population.

## 2. Materials and Methods

Study site: The shelter selected for this study was an open admission animal shelter that admitted approximately 10,000 animals annually, of which approximately 50% were cats. Both stray and owner surrendered animals were admitted. At the time of the study the shelter staff had been using the software system ^a^ for six years; however, when asked by one of the authors (Ann Therese Kommedal) the majority of the personnel reported that they had received limited training in the use of the program or in the usefulness of data collection and entry.

Software system reports: Reports from the software system ^a^ regarding daily inventory; daily intake; animal identification; location within the shelter; age; vaccination status; and URI diagnosis were collected from the study shelter using a reports generator ^c^ provided by the software manufacturer.

Manual data reports: Manual data were collected by one of the authors (Ann Therese Kommedal) observing all the cats present in the shelter during morning cleaning, between 9:30 and 11 am, every day. Data were collected 6 days a week over a 4 week period during the month of February 2009. The data points collected included daily inventory; daily intake; animal identification; location within the shelter; age; vaccination status; and URI diagnosis (Table 1). Intake date, vaccination date and vaccination status were collected from the printed software system’s cage-cards and paper medical records. A cat was recorded as vaccinated if it had a vaccine sticker and/or a handwritten note of vaccine, with or without a date of vaccination, on its cage card or medical record. Age was estimated by the observer based on size, physical appearance, presence or absence of adult canine teeth, and behavior, and animals were classified as “adult” when estimated to be 5 months of age or older, or “kitten” if estimated to be younger than 5 months based on a combination of these factors. URI was diagnosed by the observer based on the presence of any of the following clinical signs: sneezing, clear or colored nasal discharge, conjunctivitis, or ulcers/sores on the nose, lips, tongue or gums. If a cat was hiding and thus not possible to observe closely, age and URI data were not collected.

Data analysis: The software system ^a^ reports were compared to the manually collected data to assess for completeness, timeliness, availability and accuracy. All the data were transferred to spreadsheets ^b^ and evaluated for quality. Descriptive statistics were calculated for the compared data points. Sensitivity, specificity, and positive and negative predictive values were calculated for URI diagnosis entered into the software system ^a^, with the observer diagnosis as gold standard. The shelter inventory (the number of cats present in the shelter at a given time) was evaluated by comparing the inventory report generated by the software system ^a^ with the number of cats recorded by the observer on the same time and date, as well as comparing each cat’s specific location according to the software system ^a^ and manual data. The accuracy of the intake reports was evaluated by comparing the previous day’s software system ^a^ intake report with cats observed to have been admitted on the date in question. Timeliness of URI diagnosis data was evaluated by comparing the number of days after shelter entry a URI diagnosis was entered into the software system ^a^ software system *versus* the date URI was first diagnosed by the observer (Table 1 and Table 2).

**Table 1:** Data points collected by observer.

Information	Score
Intake date	
Age	0 = Kitten < 5 months
	1 = Adult > 5 months of age
	2 = Unknown, not observed
Vaccination	1 = On intake
	2 = Delayed
	3 = Prior to admission
	4 = None
	5 = Yes, date not noted
	6 = Unknown
URI status	0 = no symptoms
	1 = symptomatic
	2 = unknown/hiding
Location and inventory	0 = Animal not found in data inventory report
	1 = Animal in correct location
	2 = Animal in incorrect location
	3 = Animal not found in shelter
	4 = No paperwork

**Table 2:** Mandatory and optional data points as used at the study shelter.

Type of Data Point	Information
Mandatory	• Identification number
	• Intake date
	• Intake type
	• Species
	• Shelter location
	• Sex
	• Size
	• Outcome date
	• Outcome type
Optional	• Vaccination date
	• Age
	• URI diagnosis
	• URI resolution

## 3. Results

Two hundred and sixty cats and kittens were admitted to the shelter during the observation period, however, only 209 of these were available to be observed. An additional 108 cats were already present in the shelter at the start of the study, thus a total of 317 cats were observed during the study period. Fifty-one cats were either dead on arrival or immediately transferred to foster homes or a critical care clinic for emergency treatments. These cats were only included when comparing intake reports, while the 104 cats already residing in the shelter were excluded from analysis of intake reports. The 317 cats that could be observed in the shelter contributed a total of 2339 days of observation (care days) during the period of the study. Healthy cats and kittens contributed 1174 and 246 cat care days respectively, while 486 care days were spent on 78 cats and 24 kittens that had clinical signs of URI at some point during their shelter stay, accounting for 21% of the total cat care days. Ninety-two feral cats accounted for 433 cat care days and could not be evaluated for age or URI status.

Inventory reports: When accounting for all cats reported in the observer-collected inventory data and the software system ^a^ inventory reports, a total of 2339 cat observations were included. Ninety-five percent (2230 of 2339 care days) of data in the inventory reports were complete in that the cats were reported in the software system ^a^ inventory reports and were observed in the shelter on the same day. The incomplete data included 90 care days (4%) reported in the software system ^a^ when the cat was not observed in the shelter and 16 care days (1%) when cats were observed in the shelter but not included in the software system ^a^ inventory reports. From the complete reports 99% (2189 of 2230 data points) were accurate in that the cats were reported in the correct location in the shelter.

Intake reports: Intake data generated from the software system ^a^ reports were 77% complete (199/260 cats) when compared to observer collected data, and of these 96% were accurate. The inaccurate records included cats with an incorrect intake date or incorrect location.

Size and age: Age was not entered by shelter staff into the shelter software in the field available for this purpose. Instead, the size field was used, on the premise that “small” would represent a kitten, while medium and large would represent adult cats. Using size as a substitute for recording age resulted in a 76% accuracy compared to observer classification. The majority of cats classified as adults by the observer (172) were reported as medium or large and only 12 cats classified as adults by the observer (7%) were reported as small. However, the consistency between observer age estimates and the classification according to size was lower as only 12 kittens (as classified by the observer) were reported as small and 41 kittens as classified by the observer (77%) were reported as large or medium.

Vaccination: Both vaccination status and vaccination date reports were compared. Fifty-two percent of the software system ^a^ reports were complete as 165 from 317 cats were reported as vaccinated in both the software system ^a^ and manual data sets. Of the incomplete records five cat were manually reported as vaccinated but had no vaccination date entered in the software system ^a^; and 25 cats (8%) were of unknown vaccination status as they had no vaccine reported in the software system ^a^, on their cage card or in the medical records. One hundred twenty two cats (39%) were not vaccinated due to behavior and were noted as such on their cage cards, but had no vaccine status reported in the software system ^a^. If we were to assume that the shelter policy was to leave the vaccine status field in the software system ^a^ blank when no vaccine was given the data could have been considered as 92% complete (292/317), while the accuracy would be 90.5% (287/317). Comparisons of vaccination dates entered in the software system ^a^ and the medical records showed that 165 vaccination dates (97%) were accurately entered into the software system.

Upper respiratory infection: The overall agreement between the observer and the software system ^a^ for URI diagnosis was only 59%. Two hundred and twenty three cats were assigned a positive or negative URI diagnosis by the observer; another 92 cats were hiding and could not be evaluated. In the software system ^a^ reports cats with clinical signs of URI being incorrectly reported as healthy were twice as common as cats with an accurate URI diagnosis in the software system ^a^ (Table 3). The predictive value of the URI status in the software system ^a^ compared to observer diagnosis was below 60% both for negative and positive diagnosis (Table 4).

**Figure 1:**
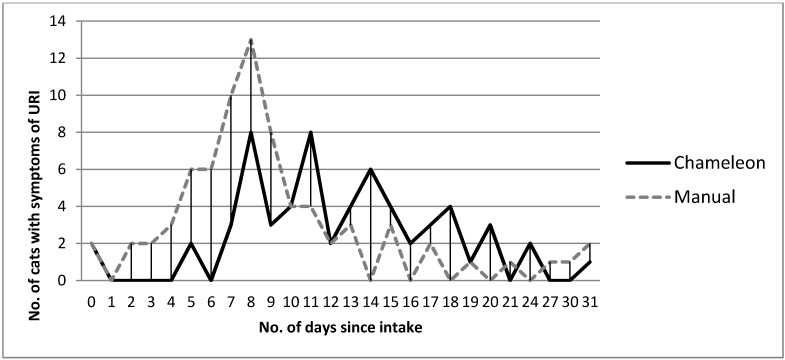
Comparing the number of days from a cat’s intake date until the onset of URI according to Chameleon and observer reports.

Timeliness: The software system ^a^ report and observer agreement when comparing the number of days from admission until diagnosis of URI was poor (Figure 1).

**Table 3:** Upper respiratory infection (URI) diagnosis in Chameleon *vs.* observed URI status.

	No. of Cats Observed Healthy	No. of Cats Observed with URI
**No. of cats reported in Chameleon as healthy**	97	68
**No. of cats reported in Chameleon as sick with URI**	24	34

**Table 4:** Measurements of reliability of URI diagnosis in Chameleon reports *vs.* observed URI status.

**Sensitivity**	0.33
**Specificity**	0.80
**Positive predictive value**	0.59
**Negative predictive value**	0.59

## 4. Discussion

Shelter medicine has many parallels to other fields of veterinary medicine dealing with animal populations, such as dairy medicine. Decades of experience in population medicine have demonstrated that herd health cannot be practiced effectively or successfully without being able to retrieve objective information about the population. Essential data include individual animal information (animal ID, sex, age, size and species), production indices of interest and animal health information (vaccination status, disease incidence, treatments, *etc.*). Herd management recommendations and decisions are most often based on the evaluation of the collected data [7,8,9].

Data collection and entry in animal shelters can pose particular challenges. Limited resources, lack of training and knowledge among shelter staff and high staff turnover are not uncommon at many shelters. These challenges can result in gaps in consistent and accurate data collection. As is seen in other herd health settings, the quality of routine data collection will likely increase if it is easily accomplished and the value is clearly demonstrated [10]. The goal of this study was to ascertain whether a shelter software system could be used to track URI frequency and selected risk factors in a shelter population, using the data that was routinely assessed, collected and entered without generating extra work for the shelter staff.

We looked at a commonly used shelter software system for animal care organizations that had been in use for several years at the study shelter. The shelter staff routinely entered both “mandatory” and “optional” data about shelter animals at the time of intake, during the animal’s shelter stay and at the time of any outcome, including data points that would be necessary to track both URI frequency and risk factors. Except for a few members of the personnel who had received extra training to be able to generate statistical reports, the majority of the shelter staff received very little training in the use of the program or the importance of data collection and entry. Potential issues with both the accuracy of the data as well as the consistency of data entry were identified.

Several studies in animal shelters and catteries have shown that the risk factors for developing URI include age, vaccination practices, hygiene protocols, length of stay and high population count [2,3,4,5]. Of these factors, reports regarding population count, age, and length of stay were easily available in the software system ^a^. URI status and vaccination date and status information when entered could be easily extracted from the inventory reports. In the study shelter the generated software system ^a^ inventory reports were 95% complete and had an accuracy of 93%. These were the reports with the highest quality and provided a good representation of the real-world situation as observed in the shelter. The most likely explanation for these reports being of higher quality than the other software system ^a^ reports is that they were audited daily during inventory rounds by animal care technicians. Although disappointing that these were the only reports that could be deemed to be of high quality, it is a good demonstration that when data points are used and audited regularly they will generate useful reports.

The intake reports provide the date of admission for each animal and form the basis of other reports including days until diagnosis of URI and length of stay. These reports also indicate the daily number of animals admitted, which can be important when planning for housing and staffing needs. In the study shelter the inventory reports were a better source of intake information than the intake reports when compared with the observer reports, again most likely due to the daily audit that was performed by the animal care technicians during morning inventory rounds. Due to the structure and data available in the inventory reports of this particular software program it is more cumbersome to use these reports to track time until onset of URI, or for planning of staffing and housing need according to expected daily admission, rather than the easily available intake reports. Thus it would be valuable to consistently enter intake information in a timely manner.

Young age has been associated with a higher risk of URI in cats housed in catteries and shelters [2,11,12]. Accurate tracking of the proportion of kittens *versus* adults is necessary to evaluate reasons for fluctuations of URI rate, as well as to plan for special housing and care needs of different age groups. There were significant disparities between observer age classification and age as recorded by staff in the shelter software which would prohibit any age-based planning or evaluation under the system in use at the time of this study. These disparities may have resulted from a combination of accuracy of initial assessment and issues with the data collection system in use at the time.

Shelter staff commonly use a combination of size/weight, physical appearance, behavior and eruption of teeth to estimate the age of animals [13]. The appearance of adult canine teeth is expected to occur at approximately five months of age and provides a practical and readily observed indicator for shelter staff to determine a cutoff point between age groups; hence this was used as the definition of kitten *versus* adults in this study. If shelter staff have insufficient training or time to accurately make age assessments, this may result in misclassification no matter how consistently data is entered and tracked. Tracking age as a risk factor for URI at the study shelter was complicated by the fact that the age field in the software system ^a^ had been made an optional data point while size was made mandatory. This was done as a result of external criticism that the shelter staff was not sufficiently trained to estimate age of kittens and thus entered incorrect age estimates. The shelter staff reported that they would enter adult cats as medium or large on the size field, while kittens were reported as small, however, there was evidently confusion about this practice. The majority of cats classified as kittens by the observer were entered as medium or large, resulting in classification as adults.

As noted, some of the disparities between age classifications may have been a result of genuine differences in assessment by the observer *versus* the shelter staff (e.g., the observer estimated the age of a cat as older than five months while the staff member entering the size estimated it as less than five months). However, most confusion regarding age is likely to arise in cats close to the five month cut off. Most cats fall well on either side of this cut off and are readily classified as adults or kittens by even casual observation. Therefore the bulk of disparity most likely arose from issues related to using the “size” field as a substitute for age. If kittens were medium or large for their age, then they may have been classified as such in the size field, while the same may have been true for the adult cats classified by shelter staff as “small”. The data would have been more useful if staff estimated age rather than using animal size as the software system ^a^ will automatically update the age field with the passage of time, and a kitten would automatically be reclassified as adult when reaching the age of 5 months. Additionally the size field could then be used correctly to record size of the animal.

Although vaccination against common respiratory agents, including feline herpesvirus and feline calicivirus, does not provide sterile protection against infection, vaccination has been shown to reduce severity and frequency of illness. It is generally recommended that animals be vaccinated immediately on intake to the shelter as a delay of even a day or two could significantly reduce the likelihood of protection in a shelter environment [14]. Therefore it may be helpful to evaluate both whether and when shelter cats receive vaccinations. Both vaccination status and vaccination date reports were evaluated in this study. Only slightly more than half of the software system ^a^ generated reports on vaccination status were complete and accurate, rendering this data point useless for evaluating this important factor. An important contributor to the poor data performance may have been that there was no option in the software system ^a^ to record a cat as not vaccinated. Assuming that a blank vaccine status field in the software system ^a^ meant that no vaccine was given resulted in a substantial increase in complete records from the 52% to 92%. Adding the option to record an animal as “not vaccinated” would thus greatly improve the usefulness of this data point.

Vaccination was part of the standard intake procedure when an animal was admitted to the study shelter; however vaccination status and date were not used as mandatory fields in this shelter. Staff members in charge of admitting animals were already required to enter animal data into the computer system, and would only need to fill out a single additional field to record vaccination status at the same time. Making intake vaccination status a mandatory field would therefore minimally increase the work load, and would facilitate better tracking of vaccination status and timing. Entering vaccination status and date would also make it possible for the shelter to schedule revaccinations as appropriate for cats and kittens and daily reports for identifying these animals could be made available.

Feline URI is a disease associated with stress and overcrowding and in general is a problem best approached from a population medicine perspective with prevention as the goal. Tracking frequency of URI is the foundation for understanding the impact of management policies, allocating resources and planning prevention and intervention strategies. Even though the study was done during the winter months, when the population at the shelter was low with few kittens relative to other times of the year, the shelter had a total of 61 cats (19%) observed with signs of URI. This resulted in 21% of the total cat care days during the study period being spent caring for cats and kittens with URI. The overall agreement between the observer and the data entered into the software system ^a^ for URI diagnosis was poor at 59%, rendering this information useless for tracking URI disease in this shelter. This disparity likely reflects both issues with assessment as well as collection of data.

Some of the disagreement can be accounted for by differences in case definition between the observer and the veterinary staff. Although the observer case definition included some mildly affected cats which would not be captured by the shelter staff’s data collection for URI, we felt it was important to include all cats with obvious signs of URI due to their clinical importance in a shelter setting. In regards to cats without signs of URI and not receiving any medical treatment that had a URI diagnosis in the software system ^a^ it is possible these were entered by staff other than the veterinary staff so that they could be evaluated for mild signs of URI that subsequently disappeared. If the URI diagnosis was not removed when the veterinary staff evaluated the animal as healthy and not in need of any treatment this would be counted as false positives. We also acknowledge that using the observer’s diagnosis as the gold standard for the URI diagnosis is problematic. The observer spent on average 1 min observing every cat on each day and it is probable that some of the cats suffering from mild or moderate URI, and reported as such in the software system ^a^, did not display any of the clinical signs during the limited time it was being observed, resulting in false negatives. The use of the URI diagnosis in the software system ^a^ for the purpose of tracking disease frequency could easily be made more useful by providing the shelter staff with a clear case definition for URI to use for diagnosis.

Time until onset of disease and time until recovery is helpful additional information to track in herd health settings. Decrease in time until onset of URI may indicate a change in pathogen/s (change in virulence or a new pathogen) or some change in the environment or management that is shortening the shelter’s “normal” interval of time to URI disease (sudden loss of staff, increase in population numbers, sudden breaks in sanitation leading to increased dose or frequency of exposure). Time until recovery is a potential indicator of severity of disease and can be a critical tool in evaluation of some management interventions, such as vaccination (use or changes in use) or changes in treatment as these strategies may have more impact by reducing disease duration and severity than by decreasing the actual number of cases. Reductions in disease duration can have a major impact on reducing costs and crowding in treatment areas.

In the study shelter a URI diagnosis was entered by the veterinary staff on the day of evaluation and start of treatment, not necessarily on the day clinical signs were first observed, and the date of resolution was inconsistently recorded. As a result these data were not useful for tracking trends over time regarding disease onset or duration. Even though the study shelter had a specifically assigned field for URI, making it possible to generate reports regarding frequency, they were not using it in a consistent manner, and did not consider the reports reliable or useful. The veterinary staff at the study shelter was using the software system to track the animals needing treatment, demonstrating that shelter staff will use data entry if it has value, e.g., generating daily treatment list. It is thus likely that they would use it for tracking disease frequency and other data if the value was clearly demonstrated and relevant reports could be easily obtained.

## 5. Conclusions

Maintaining animal health is an essential part of the stated mission for most animal shelters, yet relatively little population health monitoring occurs in shelters. Population health monitoring is essential for managing endemic shelter animal diseases such as feline URI. Without monitoring little can be known about the effects of changes made to control or prevent disease. The data entered into the software system ^a^ at the study shelter contained inaccurate data for intake date, intake age, vaccination status, vaccination date, URI diagnosis date and URI recovery date. The only report at this shelter that could have been used for population monitoring was the inventory report (95% complete and 93% accurate). Although the software system a reports generated at this shelter did not contain enough accurate data to monitor feline URI disease, it appears that all the data needed for monitoring are routinely available and in many cases were already being collected and entered—just not to the degree needed for population health monitoring. Animal ID, intake date, vaccination date, age, and daily health status should be made mandatory fields in the shelter software system to facilitate disease monitoring—a key tool needed in shelters for disease control and prevention.

## Notes

^a^: Chameleon/CMS^©^
^b^: Microsoft excel
^c^: Crystal reports
